# Supplementary material for: Are carbon emissions trading and green financial instruments synergistic? -Comprehensive quantitative research based on content analysis
Source: PLoS One. 2024 Mar 7;19(3):e0298601. doi: 10.1371/journal.pone.0298601 (PMC10919601; doi:10.1371/journal.pone.0298601)
Supplement: S1 Data — (ZIP) [file pone.0298601.s001.zip › Data/地级市Z_F工作报告.zip!/╜¡╬≈/╝¬░▓╩╨/└┤╫╘╣1⁄2╓┌║┼í╛┬φ┐╦╩2╛▌═°í┐_2013.docx]

各位代表：现在，我代表市人民政府向大会报告工作，请予审议，并请各位市政协委员和列席会议的同志提出意见。

　　一、2012年工作回顾2012年是贯彻落实市第三次党代会部署的重要一年。面对世界经济复杂多变和国内经济下行压力较大等不利因素，在省委、省政府和市委的坚强领导下，全市上下紧紧围绕建设开放繁荣秀美幸福新吉安，全力以赴抓发展、惠民生、促和谐，全市经济社会持续发展、进位赶超呈现良好势头，并获得了重大历史性机遇。八项主要经济指标迈上新台阶：预计全市生产总值迈上1000亿元（1006亿元），增长11.3%；财政总收入迈上140亿元（143.3亿元），增长21.1%；规模工业增加值迈上400亿元（402亿元），增长14.6%；服务业增加值迈上300亿元（301亿元），增长10.2%；旅游总人数迈上2000万人次（2105万人次），增长15%；井冈山机场旅客吞吐量迈上40万人次（40.2万人次），增长33.2%；城镇居民人均可支配收入迈上20000元（20150元），增长13.9%；农民人均纯收入迈上7000元（7100元），增长12.6%。五项指标增幅位居全省前列，实际利用内资和金融机构存款、贷款余额增幅均居全省第一位，全市规模工业增加值和限额以上社会消费品零售总额增幅均居全省前三位。四项指标在全省进位，工业总量前移1位，出口总量前移2位，外资增幅、内资增幅均有明显前移。吉安作为原中央苏区的重要组成部分纳入国家支持振兴发展范围，吉泰走廊成为苏区振兴的“四化”协调发展示范区和我省扶持打造的重要增长带。

　　一年来，我们重点抓了以下工作：

　　（一）大力推动产业集聚，工业经济快速增长。以工业园区为战场，以产业项目为抓手，大力开展强攻工业。工业规模与产业层次同步提升，预计全市规模工业实现主营业务收入1900亿元，增长22.4%；新增产值超亿元企业37户，累计469户；龙天勇公司在香港主板市场成功上市，实现我市企业独立上市零的突破；在改造提升传统产业的同时，加快推进战略性新兴产业发展，预计全市战略性新兴产业实现主营业务收入980亿元，增长25.1%，其中电子信息产业实现320亿元，占全省四分之一强。工业园区建设快速推进，完成基础设施投入43亿元，建成标准厂房220万平方米，预计实现主营业务收入1660亿元、税收70亿元，分别增长24%和16%。围绕昂起龙头，推进井开区完善提升西区、拓展建设东区，实现主营业务收入400亿元，增长44.7%。吉安县、泰和县2家工业园区被列为首批省级重点工业园区，新增吉州、青原、吉水、安福、永新5家百亿园区，总数达到10家。

　　吉泰走廊产业集聚效益明显，区域内规模工业实现增加值220亿元、主营业务收入1180亿元，分别占全市规模工业的54.7%和62.1%。

　　（二）着力培育新的增长点，现代服务业繁　　荣发展。坚持工业和服务业双轮驱动、多点发力，促进现代服务业做大做强，全市服务业实现地方税收31.3亿元，增长28.3%。形成了2个省级服务业集聚区和4个省级龙头企业，总数居全省第二。商贸物流业加速发展，全市城区在建规模以上市场项目34个，市中心城区十大市场体系18个项目完成投资29.7亿元；一中心、一平台、六园区现代物流体系建设加快推进。市场消费有效扩大，预计全市实现社会消费品零售总额270亿元，增长18.1%。旅游业保持旺盛势头，“三山”战略扎实推进，全市实现旅游总收入165亿元，增长32.9%。金融业快速发展，全市金融机构各项存款余额1285.1亿元，增长22.6%，贷款余额567.5亿元，增长26.6%。

　　（三）夯实农业基础地位，农业经济稳步发展。认真落实强农惠农政策，加强农业基础建设，强化耕地保护，抓好农业生产，农业经济保持稳产增效良好态势。主要农产品产量稳步增长，粮食生产实现九年连增，总产达386.7万吨；肉类总产52.3万吨，家禽出笼9000万羽，水产品总产19.6万吨，均有增长。特色产业快速发展，全市新增井冈蜜柚6.6万亩、高产油茶11.1万亩、绿色蔬菜5万亩、花卉苗木5.2万亩、珍贵楠木6.8万亩、有机茶叶2.8万亩，永丰、新干、峡江被列为全国蔬菜生产重点县。农业产业化进程加快，全市新增农村土地流转面积46.6万亩，新增市级龙头企业25家、省级龙头企业19家，总数分别达到271家和62家；新增农民专业合作社484个，达到2080个。农田水利建设稳步推进，投入资金17.4亿元，加固水库206座、堤防54公里，建成高标准农田13.3万亩，改善灌溉面积50万亩，农业综合生产能力进一步提升。

　　（四）统筹推进城乡建设，城乡发展一体化进程加快。围绕建设大气雅气秀气吉安，大力推进城建重点项目攻坚战和美丽乡村建设大会战。全市实施重点城建项目231个，完成投资150亿元；新增城镇建成区面积27.2平方公里，城镇化率提高2个百分点，达到41.6%。吉泰城镇群建设加快推进，吉泰走廊“1+8”规划体系初步形成,公交一体化取得实效。中心城区组建庐陵新区，规划樟山新区和青原山对接新区，重点推进了120个城建项目，完成投资80亿元，增长30.6%，庐陵民俗园、滨江内湖景观工程等项目竣工，科技馆与青少年活动中心、博物馆和城市展示馆等一批公建项目完成主体工程，古后河绿廊、螺湖湾湿地公园、赣西堤及周边　　 棚户区改造、全民健身体育中心等项目正加快建设，新增建成区面积4平方公里、达到63.6平方公里，新增公园和园林绿地面积95.5万平方米。县城建设完成投资70亿元，新增建成区面积16平方公里。小城镇重点推进了31个示范镇建设，完成投资20.5亿元，新增建成区面积7.2平方公里。美丽乡村重点推进了995个村点建设，并由示范建点向集中建带迈进，涌现了杨万线等一批美丽乡村综合示范带。城镇管理进一步加强，在中心城区大力推进“四城同创”、城乡洁净工程、无物业小区改造、市容市貌专项整治和闲置土地清理；在县市城区实施美化亮化、园林绿化、老城区改造提升和城区管护工程，井冈山市再获国家卫生城市；在农村加强对农民违章建房的整治，对主要干道沿线村庄进行庐陵建筑风格改造提升，有效改善了城乡面貌。

　　（五）坚持项目带动战略，有效投入持续扩张。加强重大项目的开发立项、资金筹措、土地供应和协调推进，投资保持较快增长，预计全市完成固定资产投资961亿元，增长30%。争资争项再创历史新高，一批重大项目纳入苏区振兴规划，全市争取国家和省支持的项目资金27.46亿元，增长76.5%；争取省级预留建设用地指标1.25万亩，增加1.8倍。项目建设加快推进，开工重点项目328个，竣工40个。105国道中心城区改道暨永和连心大桥、青东公路二期等项　　目进展顺利，全市新增农村公路662公里，行政村客运班线通达率为93%。大力配合国家、省重点工程建设，京九铁路电气化改造工程竣工，峡江水利枢纽实现大江截流，赣江石虎塘航电枢纽3台机组完成并网发电调试；抚吉高速、吉莲高速吉安境内建成通车，全市新增高速公路162公里，实现县县通高速。大力推动全民创业，召开动员大会、制定激励政策、出台实施意见，全市新增私营企业2807户、个体工商户19599户，非国有投资占全市固定资产投资的77.4%。

　　（六）全面推进对外开放，开放型经济水平明显提升。坚持实施大开放主战略，加大对台招商力度，突出招大引强。预计全市实际引进内资319亿元，增长28.4%；实际利用外资5.65亿美元，增长12.4%；现汇进资2.1亿美元，增长91.4%。拓展招商领域，在突出工业招商的同时，拓展农业、服务业对外开放，全年新引进5000万元以上项目226个，其中战略性新兴产业项目112个，现代服务业项目25个，绿色食品加工和现代农业产业项目15个。积极探索板块对接、产业链对接新模式，推进了深圳南山示范园、香港六星产业园、台湾德昌产业园开发建设。积极开拓国际市场，全年出口25.7亿美元，增长24.3%。

　　（七）坚持民生为重，社会保障更加有力。

　　全市各级财政用于民生支出175亿元，比上年增加29亿元。实施积极的就业政策，发放创业贷款8.4亿元，创业培训7837人，新增城镇就业9.3万人。实现城乡居民社会养老保险全覆盖，参保人数210.4万人，增长23.8%；提高了城乡居民最低生活保障水平；开工建设保障性住房1.55万套、建成9116套，完成棚户区改造2250户、农村危房改造1.64万户；争取上级救灾资金5000万元，减灾救灾成效明显；农村安全饮水工程扎实推进，解决了38.8万农村居民和6万名农村师生安全饮水问题。新一轮扶贫开发全面展开，实施整村推进扶贫项目2062个，完成移民扶贫搬迁1.4万人。峡江水利枢纽6680户移民新居建设有序推进。生态环境持续改善，全市完成造林39万亩，城镇绿化和城市“绿肺”建设4万亩；井冈山国家级自然保护区被联合国教科文组织批准为“世界生物圈保护区”。大力开展环境保护，整改存在环境问题企业665家，关停不符合产业政策和布局不合理的企业198家。加强和创新社会管理，推进12个社会管理重点项目建设和社区网格化管理，全面开展清访行动、安全生产集中整治、社会治安专项整治，公众安全感指数高于全省平均水平。

　　（八）坚持统筹协调发展，各项事业全面进步。科技创新能力增强，全市获国家和省级科技项目67项，开发省重点新产品55项，永新县成为首批科技惠民计划试点县。教育事业稳步推进，完成教育投入47亿元，增长23.5%；市特教学校和7所思源学校投入使用，新建和改造农村义务教育学校332所；全市高考二本以上上线率在全省排名前移2位；职业教育加快发展，吉安职业技术学院动工兴建；济困助学蔚然成风，师德师风建设成效显著。文化事业不断繁荣，全市11个国有文艺院团全部完成转企改制，市文化艺术中心大剧院成功托管，全市“三馆一站”全部实现免费开放，丰富了人民群众文化生活。医药卫生体制改革深入推进，全市2511所村卫生室全部实施国家基本药物制度。食品药品安全态势平稳。低生育水平持续稳定。全民健身运动广泛开展。驻吉部队和民兵预备役人员积极承担急难险重任务。国家安全、国防动员、拥军优属、人民防空等工作进一步加强，统计、物价、审计、供销、方志、档案、民族宗教、外事侨务等工作取得新成绩，妇女儿童、社会慈善、残疾人及老龄事业有了新发展，供电、工商、质监、气象、水文、海事、烟草、盐务、石油、机场、海关、检验检疫、高速路政、邮政通信等中央和省属单位服务地方经济社会发展作出了新贡献。

　　过去一年，政府自身建设得到有效加强。

　　依法行政水平有所提高，自觉接受市人大的依法监督和市政协的民主监督，坚持向市人大报告工作和向市政协通报工作制度，认真听取人大代表和政协委员的意见建议，全年办理人大代表意见建议140件，办结率为100%、满意率为97.9%;办理政协提案196件，办结率为100%，满意率为98.4%。努力提升行政效能，精简市级非行政许可审批项目52项，精简30.6%。进一步加强廉政建设，全面落实党风廉政建设责任制，加强行政权力运行监督，加大项目投资评审力度。深入开展“三大”主题教育暨集中整治影响发展环境的干部作风突出问题活动，严格实行“六个不准、六个一律”，机关效能和公务人员服务意识明显增强，群众对干部作风的满意度达96%，比上年提升10个百分点。开展“市长·市民面对面”活动，全面落实领导干部带头接访下访制度，认真受理办复群众来信来电1.4万件，有效解决人民群众的切身利益问题。

　　各位代表，过去一年的成绩来之不易。这是省委、省政府亲切关怀和市委正确领导的结果，是市人大、市政协及社会各界有效监督和大力支持的结果，是全市人民团结拼搏、奋发进取的结果。在此，我代表市人民政府，向全市广大工人、农民、知识分子、干部和历任老领导、老同志，向各民主党派、工商联、无党派人士和社会团体，向驻吉人民解放军、武警官兵和公安消防干警，向中央和省驻吉单位，向所有关心、支持和参与吉安建设的同志们、朋友们和社会各界人士，表示衷心的感谢并致以崇高的敬意！在肯定成绩的同时，我们也清醒认识到经济社会发展还存在不少困难和问题，主要表现在：经济总量偏小，人均水平较低，欠发达的基本市情仍未根本改变；城镇化水平偏低，扩大城市人口、繁荣城市经济的任务还十分艰巨；农业农村基础设施还较薄弱，农业产业化水平偏低，农民持续增收的压力较大；土地、资金、用工等要素制约仍然严峻，部分企业经营困难；社会保障水平还不高，保障和改善民生特别是与全国人民同步实现小康目标的任务非常艰巨；政务环境还有待进一步改善，政府行政水平、行政效能和公共服务能力还不能完全适应群众和发展的需要。对此，我们一定高度重视，并切实加以解决。

　　二、2013年的工作任务2013年是深入贯彻落实十八大精神的开局之年，是全面推进中央苏区振兴、打造吉泰走廊重要增长带，为全面建成小康社会奠定坚实基础的一年。新的一年，尽管我们面临国际经济复杂多变、区域竞争日趋激烈、资源环境约束趋紧等困难和挑战，但我们仍处于大有作为的重要战略机遇期，加快发展的有利条件多于不利因素。在国际经济的转型调整中，我们仍然可以捕捉到扩大开放、借助外力新机会。国家实施积极的财政政策和稳健的货币政策，实施结构性减税，加大投入力度，增加信贷总量，有利于我们加快产业发展；特别是中央苏区振兴发展战略的实施和吉泰走廊发展战略的升级，为我们提供了前所未有的多重叠加政策机遇。近年来我市一批重大项目的建成投产，为经济社会发展提供了有力支撑；全市上下风清气正，团结奋斗、合力共为的氛围浓厚，尤其党的十八大提出全面建成小康社会的宏伟目标，正激励全市上下顽强拼搏、奋发进取。只要我们坚定信心，抢抓机遇，扎实苦干，就一定能推动经济社会发展再上新台阶。

　　2013年政府工作的总体要求是：深入贯彻落实党的十八大和中央、全省经济工作会议精神，按照市第三次党代会部署，坚持以科学发展观为指导，紧紧围绕“五位一体”总布局，牢牢把握中央苏区振兴和吉泰走廊建设的历史性机遇，以提高经济增长质量和效益为中心，坚定不移地主攻项目、决战两区，做大总量、加快集聚，坚定不移地推进新型工业化、信息化、城镇化、农业农村现代化“四化”同步互动，“三产”共生演进，坚定不移地改善民生、促进社会和谐，开拓创新，真抓实干，保持经济持续健康较快发展，努力为建设开放繁荣秀美幸福新吉安、全面建成小康社会奠定坚实基础。

　　按照上述要求，在总体目标任务上力求实现“两个略高于”，即主要经济指标增幅略高于全国全省平均水平，略高于全面小康年度目标要求；“两个占比提高”，即财政总收入占GDP比重和税收占财政收入的比重分别比上年提高1个百分点以上；“两个同步”，即居民收入增长与经济发展保持同步，农民人均纯收入增长优于城镇居民可支配收入增长或保持同步。2013年全市经济社会发展的主要预期目标是：生产总值增长11%以上，其中，规模工业增加值增长16%；财政总收入增长18%；固定资产投资增长25%；社会消费品零售总额增长16%；外贸出口总额增长10%，实际利用省外资金增长15%，实际利用外资增长10%；城镇居民人均可支配收入增长12%，农民人均纯收入增长12.7%；城镇化率提高2个百分点；人口自然增长率控制在7.5‰以内；单位生产总值能耗下降3.2%，化学需氧量、二氧化硫、氮氧化物排放量分别下降1%，氨氮排放量下降1.2%。

　　在实施过程中，我们将坚持稳中求进、好中求快，在提高质量和效益的基础上，力争经济发展速度更快一些，群众所得实惠更多一些，资源和环境指标控制得更好一些。

　　围绕上述目标，重点抓好以下八个方面的工作。

　　（一）突出重点，聚集力量，打造重要增长带。紧紧抓住吉泰走廊战略升级的历史性机遇，围绕将吉泰走廊建设成“四化”协调发展示范区和产城互动、城乡一体、充满活力的重要增长带，大胆先行先试，创新体制机制，完成规划、加强引导，强化考核、落实责任，使政策落地、投入加大、项目加快推进，力争区域内生产总值增长13%；城镇化率提高3个百分点，达到56%；规模工业增加值增长18%，总量占全市的比重达到57%。

　　加快形成产业发展整体优势。以井开区为龙头，以电子信息产业为主导，主攻整机、带动配套、链式发展，构建电子信息产业的组合优势，打造完整的产业体系，力争区域电子信息产业完成主营业务收入300亿元；错位发展生物医药、先进装备制造等产业，实现各园区优势互补、相互配套。围绕千亿园区目标，推进井开区东区开发，完成供水、供电、道路、跨铁路大桥等基础设施，加快企业孵化中心和产业板块建设；提升西区产业集聚水平，增强产业投资密度，推动现有企业再投资、再开发。加快共享平台建设，完善“无水港”功能，出口加工区封关运行，加快口岸作业区建设，争取开通“五定”班列，建成吉水电镀集控中心，实现区域内乃至更大范围共用共享。

　　加快推进产城融合发展。按照“核心带动、两翼联动、带状发展”的原则，加快“一核两翼三带”城镇群建设。着眼打造核心区，加速井开区、吉安县城融城步伐，加快构建“大吉安”城市格局；推进以吉水、泰和为两翼的副中心建设，支持两个县城拓展规模、完善功能、提升品位；打造沿105国道、朱北线、樟吉（大广）高速三条城镇带，在继续抓好沿线已定10个卫星镇建设的同时，加快醪桥、樟山、曲濑、石山、南溪开发建设，推动吉泰走廊小城镇串珠成链、带状集聚。推进生态布局与城市建设相配套，居住区域和产业项目相协调，新镇开发建设与老街改造提升相结合，促进生产要素和人口有序集聚。加快建设区域内服务居民生活、服务产业发展的市场体系，建成劳动力市场和人才市场，实现区域内市场网络互联互通。

　　探索农业转移人口市民化的有效途径，推动人口向吉泰走廊城镇群集中。

　　加快构建城乡一体格局。推动供水、供气、供电、公共交通、邮政等基础设施在区域内的一体化建设。完成中心城区水厂扩建，实现中心城区供水与吉安县、井开区供水管网的互联互通，推进吉水县、吉安县、泰和县供水管网向周边乡镇延伸。以中心城区为平台，加快供气设施向周边县乡延伸，实现井开区、吉安县城和凤凰镇全面通气，争取天然气接入吉水、泰和县城和天玉镇。进一步完善区域内公共交通，实现区域交通大循环。加快县城和园区污水处理设施建设，实现吉水、泰和工业园区工业污水、生活污水处理设施建设和营运。

　　加快“数字吉安”和“光网城市”建设，推进互联网进村入社区步伐，完成中心城区和各个重点镇的入户任务，并覆盖到95%以上乡村。实施区域内公共服务提升工程，推进教育、卫生等公共服务设施均等化。

　　（二）加快集聚，提升水平，加速新型工业化。以　　 提高增长质量和效益为中心，坚持传统产业与新兴产业融合式发展，在转变发展方式、优化结构的进程中，促进工业上规模、上能级、上水平，实现规模工业增加值475亿元。

　　培育壮大战略性新兴产业。重点围绕电子信息、生物医药、先进装备制造、新能源及节能技术、新材料等产业，加快推进重大项目建设，推动产品终端化、品牌化、集成化。推进柏兆计算机主板项目、天人生态微生物农药新技术开发，完善普正药业总部基地扩产改造工程，启动杰克机床扩建提能、电线电缆厂特种电缆和配套材料建设，加快台湾德昌产业园建设，积极引进长宜新能源等项目。力争开工建设战略性新兴产业亿元项目80个以上，竣工投产60个，全市战略性新兴产业主营业务收入达到1200亿元，增长25%。

　　改造提升传统产业。着力振兴酒业、提振烟业、提升茶业、做大食品加工业。加快堆花酒厂入园扩建、提能扩产，推进井冈红米酒、勃客保健酒、燕京啤酒、赣泉啤酒、泰和乌鸡酒扩大市场、带动生产，推进葡萄酒开发生产。着力抓好井冈山卷烟厂易地改造扩建，按照30万标箱产能，年内尽快动工兴建。积极推进安福火腿、遂川板鸭等产品的营销扩产步伐，推进泰和乌鸡系列开发。加快冶金建材产业改造提升，推动思瑞铜业等铜加工企业发展精深加工，推进南方水泥等新型干法生产线达产达标。

　　扶持壮大成长型企业。加大企业帮扶力度，通过银企对接、协调招工、政策扶持，促进小微企业发展，扶持企业成长壮大，力争新增规模企业50家、税收上千万元企业10家以上。开拓产业发展新领域，大力培育建筑业、印刷业和文化产业，形成一批新兴企业。支持企业加快上市步伐，力争年内完成1家企业上市、3家企业股改，重点培育10家上市后备企业。

　　推进园区做优做强。注重全市工业园区的规划编制和产业布局，突出主业、板块聚集、链式发展，打造通讯终端、机电液压、林产、食品、五金、皮革、盐化等一批特色产业基地。推进园区配套攻坚，完成标准厂房150万平方米，加快道路管网延伸扩容。着力促进招工稳工，实施园区安居工程，建成家庭公寓1500套。推进区内建设和就近城区配套相结合，完善培训教育、技术创新、创业孵化、融资担保、生活娱乐等服务保障，提升园区综合竞争力，力争年内所有工业园区主营业务收入过百亿元。

　　（三）统筹兼顾，建管并重，加快城乡发展步伐。

　　坚持把庐陵文化元素渗透到城乡建设中，加快建设大气雅气秀气吉安，完善功能、提升品质、优化环境，实现城乡同建同治。

　　加快中心城区建设。坚持精心规划、精致建设、精细管理，打造精品城市。完善中心城区规划，拓展规划范围，深化规划设计，以控制性详规和指标设计引导土地出让、建筑设计和项目建设。中心城区重点推进165个城建项目，年内完成投资118亿元。继续拓展城市框架，加快形成城市外环线，推进庐陵新区、樟山新区、青原山对接新区、城西片区建设，拓展建成区面积5.5平方公里。完善城市功能，完成城南文化行政中心公建设施建设，年内全面投入使用；加快城南商业慢行系统、庐陵国际酒店、全民健身体育中心建设，启动河东滨江会展城市综合体、城西综合商城建设，加快赣西堤及周边棚户区改造、古后河绿廊、螺湖湾湿地公园等工程建设。推进老城区改造提升，抓好小街小巷和老住宅区综合改造，实施棚户区改造2700户。深入推进“四城同创”，加快中心城区公共服务设施建设，新建和改造40座公厕和垃圾中转站；抓好背街小巷整治，用三年时间完成“城中村”改造任务。强化物业管理，确保新建小区物管覆盖率达到100%，老住宅小区达到90%。

　　建设城市数字化管理平台，提升城市管理的时效性和精细化水平。

　　推进城镇建设。加快县城扩展充实新区、改造提升老城，进一步实施路网建设提升、旧街改造整治、绿化拓展延伸，推进县城创建省级森林城市；健全环境卫生长效管理机制，推进所有县城卫生城市创建升级活动。

　　全面推进小城镇建设，以省市示范镇建设提升，带动各小城镇完善公共设施，加快改造整治，健全管理机制，确保两年内214个乡镇全面完成整治，建设一批具有较强带动能力和庐陵特色的中心镇。强化产业支撑，完善配套政策，加速人口向城镇聚集。

　　深化美丽乡村建设。按照全域规划、整体布局、镇村联动、带状推进的思路，以高速公路、铁路、国省道和吉泰走廊沿线村庄为重点，坚持“五美”标准和“八不八多”原则，突出庐陵风格，深入推进910个村点建设，每个县（市、区）建设一条以上美丽乡村示范带。

　　坚持抓产业促发展，大力发展特色产业和富民工程；深入开展农村环境治理，完善提升庭院绿化、道路绿化、村旁绿化。全面启动乡村规划，实现30户以上自然村规划全覆盖，落实“八个禁止”，坚决整治“两违”建房。

　　加快城乡基础设施建设。开工建设和建成一批基础设施，提高城乡发展的支撑能力。加快105国道中心城区改道暨永和连心大桥建设，实现年内竣工通车；启动城北庐陵大桥建设；力争吉安绕城高速、新干至东乡高速开工建设；配合推进井睦高速、衡茶吉铁路，实现年内竣工；积极争取和配合昌吉赣客运专线、岳阳－吉安铁路年内开工；加快推进井冈山机场扩建工程、朱北线改造、吉水县城市防洪路堤结合工程等项目；新干、遂川县高速公路连接线建成通车；完成800公里农村公路建设任务。开工建设井冈山水电站、新干航电枢纽、遂川清秀风电、泰和水槎风电等项目；配合推进峡江水利枢纽工程、西气东输三线工程等项目，实现赣江石虎塘航电枢纽工程竣工投产。

　　（四）突出特色，提高效益，推进农业现代化。加快现代农业示范区建设，大力发展特色产业，提高农业产业化水平，用先进技术装备农业，加快推进传统农业向现代农业转变。

　　大力发展特色产业。严格保护耕地，确保粮食播种面积960万亩，稳步发展畜牧、水产业。调优农业结构，大力发展井冈蜜柚、高产油茶、绿色蔬菜、花卉苗木、珍贵楠木、有机茶叶六大特色产业。实施井冈蜜柚"百千万"工程，打造5个万亩示范基地、10个千亩示范片和30个以上百亩示范点，新增面积8万亩。抓好25个蔬菜标准园和13个标准化生产示范基地建设，新增面积2.1万亩。整合林业项目资金，推进规模化、标准化示范基地建设，新增高产油茶11万亩、花卉苗木4万亩、珍贵楠木8万亩。加快发展有机茶叶，新增面积2.5万亩。

　　推进规模化经营。在切实保护农民利益的前提下，加大土地流转力度，重点推进荒山荒地、稀疏残次林地和撂荒土地的流转，扶持规模化经营。大力发展开放型农业，培育农业产业化龙头企业，促进产加销联动，力争培育年销售收入超亿元企业35家、超5亿元企业6家、超10亿元企业2家。扶持农民专业合作社发展，力争新增200个，带动农民入社4.3万人。

　　加快农技推广。抓好井冈山国家农林科技园建设，推进综合服务中心、花卉苗木、特色果蔬、休闲度假、农产品加工示范区“一心四区”建设，每个县（市、区）至少建设一个现代农业示范园，带动全市设施农业、休闲农业、高效农业发展。加快农业科技推广应用、成果转化和机械化推广，提高农业生产的比较效益。

　　夯实农业基础。整合资金，优先向赣粤、泰井高速和吉泰走廊105国道沿线布局，集中连片、整体开发，新建高标准农田18万亩。抓好农村水利工程，除险加固225座小（二）型水库，推进南车灌区续建工程，积极争取白云山水库和万安水库灌区工程早日实施，逐步完善末级渠系和田间工程配套，力争新增、恢复灌溉面积10万亩，改善灌溉面积20万亩。加快农业气象服务、动物防疫、植物病虫害防治体系建设。

　　（五）扩充总量，提升层次，加快发展现代服务业。

　　坚持工业和服务业双轮驱动，打造现代服务业集聚区，培育龙头企业，推进重点项目，加快服务业发展提速、总量扩充、水平提高，力争全市服务业实现增加值345亿元，增长11.2%。

　　加快发展旅游业。积极创建国家旅游扶贫试验区，以井冈山为龙头，大力推进“三山一江”战略，加快旅游产业发展，确保全年接待国内外游客突破2500万人次、旅游总收入突破200亿元。推进井冈山世界“双遗产”申报和旅游上市工作，加快罗浮片区规划建设；加快武功山景区开发，实现武功山金顶索道开通营运、羊狮慕景区建成开放；推进青原山整体开发，完成阳明书院旧居旧址维修、吉青公路改造提升等工程。启动开发万安湖、石虎塘、峡江水利枢纽等赣江库区旅游项目。

　　同时，加快中心城区和周边县区旅游资源的统筹融合，以庐陵文化生态园为依托，建设市中心城区旅游服务中心，实施景区套票制度，实现联合经营。推进吉州窑遗址公园、白鹭洲书院公园、古后河绿廊、螺湖湾湿地公园的旅游开发。

　　繁荣商贸物流业。培育中心城区商贸集聚区，吉安农副产品交易中心、城南专业市场、天虹商城年内竣工投入使用；加快高档家装家私市场、吉福路小商品批发市场、庐陵美食城、儒林里特色古街、庐陵文化商业街等专业市场建设步伐。积极引进电子商务企业，扶持企业开展电子商务营销，争取列入全国电子商务示范基地。积极推进“万村千乡”市场工程，提升改造乡镇农贸市场，培育农村流通合作服务组织。打好“全国公路运输枢纽城市”品牌，构建以井开区综合物流中心为龙头的网络化物流体系，建设青原区北、吉水城南、峡江河西、新干河西、吉州城西、遂川东区、永新7个物流园区，建成井开区、泰和县、吉安县、峡江县、吉水八都、新干城北6个物流园区。

　　培育壮大文化产业。发展壮大印刷复制、动漫创意、演艺娱乐、广播影视、文化旅游和节庆会展六大产业，加快形成文化产业集聚效应。加快吉安市印刷产业基地、中国动漫（吉安）体验中心、华影时代电影城和中国·渼陂美术写生基地等项目实施进度，争取早日运行。发挥文化艺术中心大剧院托管机制优势，推出一批精品演艺节目，丰富市民文化生活。搞活采茶歌舞剧院运作机制，创作生产一批精品节目，展示红色文化，开发庐陵文化特色产品。

　　发展金融保险业。争取交通银行等商业银行落户吉安，支持各商业银行在各县（市、区）设立分支机构。

　　健全融资担保体系，建立市本级信用担保公司，实现各县（市、区）担保企业增资扩股。进一步加强银企对接，力争全年新增贷款120亿元以上，存贷比明显提高。推进金庐陵公司完成债券发行，市城投公司做好新一期债券发行准备。拓展各种融资渠道，开发运用多种金融产品，支撑产业发展。

　　（六）深化改革，扩大开放，增强经济发展动力和活力。坚持改革不停顿、开放不止步，推进体制机制创新，加快重要领域和关键环节改革步伐，拓展开放广度和深度，为全市科学发展、进位赶超提供动力活力。

　　深入推进重点改革。创新体制机制，加强目标管理考评考核，建立有利于项目推进和攻坚克难的机制。加强重大资金的投资评审和决算审计，确保资金有效运行和规范使用。按要求对事业单位进行分类，做好事业单位改革准备。深化国有林场改革，推动林业专业生产合作社改革试点，加快现代集体林权建设。推进医药卫生体制改革，加大国家基本药物制度执行力度，加快推进公立医院改革，巩固完善新农合制度。深化财政体制改革，将国库集中支付改革延伸到县级财政，全面推开县级部门预算。进一步简政放权，促进富县强镇，激发县乡发展的潜力活力。

　　全面扩大对外开放。建立招商责任推进机制，落实奖励激励政策。坚持全方位开放，实现工业、城建、现代服务业、现代农业宽领域招商，更加注重抱团式引进、板块式承接，加强产业配套。加大招大引强引优力度，巩固长三角、珠三角、闽东南的招商成效，突出境外招商，着力开展对台招商，努力打造“江西的昆山”。

　　进一步优化出口结构，扩大生产性企业自营出口，提高机电产品、高新技术产品的出口比重，建设吉州区、吉安县、新干县三个省级出口基地，打造吉安市服务外包示范园。

　　大力推进全民创业。培育创业主体，广泛动员和鼓励城乡群众、干部职工投身创业。积极吸引在外务工人员、经商人员、企业家回乡创业。推进企业二次创业，实现本土企业优强化。搭建创业平台，依托城镇街区、工业园区，积极创办创业一条街、创业孵化区。强化政策扶持，支持实体经济发展，放宽创业门槛。加大对能人创企业、小企创大业的扶持力度，推动县域经济发展壮大，实现半数以上县（市、区）财政收入过10亿元，3个县过15亿元，1个县迈上20亿元。

　　（七）以人为本，改善民生，加强社会建设。坚持发展成果更多更公平地惠及广大群众，加快群众致富步伐，发展各项社会事业，积极化解各类矛盾，提升群众幸福感。

　　切实保障和改善民生。继续实施积极的就业政策，重点帮助“零就业家庭”、“4050”人员、失地农民等困难群体优先就业，新增城镇就业9.7万人。完善覆盖城乡居民的养老保险体系，提高城乡低保、农村“五保”、困难群众大病救助等社会救助水平，每个县（市、区）规划建设一所高标准社会福利中心。加快推进保障性安居工程，开工建设保障性住房和棚户区改造2.66万套，实施农村危旧房改造9.18万户。抓好农村饮水安全工程，解决35万人安全饮水问题。大力推进罗霄山脉片区连片扶贫开发，抓好省市帮扶“四个一”扶贫工程，落实661个贫困村整村推进，完成1万名以上移民搬迁扶贫任务。全面完成峡江水利枢纽移民安置，积极扶持移民新村产业发展，努力实现移民“搬得出、稳得住、富得起、不反复”的目标。

　　大力发展社会事业。加快发展教育事业，新建37所乡镇公办中心幼儿园，利用闲置校舍改造154所农村幼儿园，实施农村初中校舍改造和边远艰苦地区农村教师周转宿舍建设工程，加快吉安职业技术学院、白鹭洲中学新校区和吉安一中新校区建设，提升优质资源办学规模。大力发展文化事业，加强基层公共文化服务设施建设，市博物馆和城市展示馆、市科技馆和青少年活动中心、市档案馆文件查询中心年内开放。

　　推进卫生事业发展，加快市中心医院城南分院、市儿童医院、吉水和遂川县医院、永丰中医院建设；推进家庭医生进社区。积极推动科技协同创新，深入实施科技创新“六个一”工程和科技惠民计划。加强人口计生工作，加大出生人口性别比整治力度。组织办好市第四届运动会。

　　加强和创新社会管理。强化基层社会管理服务平台建设，实行社区网格化管理，构建政府、社区、企业、社会组织和公民联动的社会管理机制。切实加强源头预防，健全社会稳定风险评估机制。积极做好新形势下的群众工作，健全维护群众权益机制。创新信访工作机制，建立市县乡村四级矛盾纠纷联动化解平台，妥善解决群众合理合法诉求。加大互联网监管力度，加强引导网络舆情。积极推进安全生产标准化建设，重点抓好生产企业、各类学校、道路交通和高危行业的安全管理。

　　开展质量兴市活动，积极创建食品药品安全示范区。推进国防和后备力量建设，做好国防教育、拥军优属和兵役工作。加强应急管理工作，健全社会预警体系和应急机制，不断提高突发事件应急处置和保障公共安全的能力。强化社会管理综合治理，严密防范和依法打击各类违法犯罪活动，促进社会平安稳定。

　　（八）保护环境，建养生态，提升绿色生态核心竞争力。坚持经济与生态齐抓、发展与环境共赢之路，把生态文明建设融入经济社会建设各方面和全过程。

　　推进节能减排。深入实施节能重点工程，抓好有色、冶金、建材等重点行业节能技术改造，切实提高高耗能行业能源利用水平，全市万元工业增加值能耗下降5%。开展资源综合利用，推进铁尾矿资源综合利用。

　　加快再生资源回收利用体系建设，争取列入全国再生资源回收利用体系建设试点城市。加强化工、冶金、建材等重点行业废气、废渣、废水处理设施建设，城市环境空气质量稳定在二级以上。实施井开区循环化改造，推进水资源循环综合利用和电子线路板、电子废弃物、中药提取废渣、废铅酸蓄电池回收加工和综合利用，创建国家级生态工业园。

　　加强环境保护。深入开展工业园区污染集中治理，重点加大对化工、造纸和重金属企业污染治理。加大饮用水源保护力度，强化水源地、城市取水口等重点地区的环境保护，确保集中式饮用水源水质100%达标。开展城区油烟、噪声、污水及管网配套设施综合治理，抓好小（二）型以上水库和农村规模化畜禽养殖污染防治，大力推进以生活污水、生活垃圾和面源污染等治理为重点的农村环境综合整治，不断改善城乡人居环境。

　　加强生态建养。以创建国家森林城市为抓手，实施“森林城乡、绿色通道”建设，重点建设城市片林和森林公园，突出抓好以高速公路、国省道为重点的通道绿化提升工程，大力发展林下经济，完成造林任务37.9万亩。加大赣江流域生态保护区和国家级、省级自然保护区保护力度，启动万安库区湖泊生态环境保护试点。

　　加强野生动物的保护，在猎捕、交易、餐饮等环节加强整治和管理。实施11条中小河流综合治理工程，加快推进水土保持工程建设。支持井冈山创建国家级生态市，积极开展省级生态县、生态乡镇、生态村示范创建活动，提升绿色生态品牌优势。

　　各位代表，新的形势和任务，对政府工作提出了新的更高要求，而我们的一些领导干部和工作人员与发展的要求和群众的期待相比，还有不小的差距。有些部门管理不严、工作不力、懒散松懈；少数干部精神懈怠、作风漂浮、能力不足，形式主义、官僚主义等问题仍不同程度地存在，有的甚至以权谋私、贪污腐败。对于这些问题，我们一定高度警醒，并尽最大努力予以解决。

　　我们将着力建设法治政府、服务政府、廉洁政府，始终把人民满意作为政府建设的追求目标。

　　全面坚持依法行政。严格按照法定权限和程序行使职权、履行职责，做到依法决策、依法行政。主动接受人大的法律监督、工作监督和政协的民主监督，认真办理人大代表意见建议和政协提案，认真听取民主党派、工商联、无党派人士和各人民团体的意见。进一步完善公众参与、专家论证和集体讨论相结合的决策制度，做到问政于民、问计于民。继续完善政务公开、新闻发布、社会公示和听证制度，自觉接受舆论监督和社会监督，让权力在阳光下运行。

　　不断提高行政效能。进一步精简行政审批项目，缩短审批时限，优化审批程序，提高办事效率。打造便民服务平台，实现市、县网上审批、电子监察及公共资源网上交易系统互通互联。坚持领导干部深入一线、靠前指挥、攻坚克难，对重点产业、重大项目实行挂点帮扶、现场督查，完善工作推进机制，不断提升政府推进科学发展、进位赶超的执行力和操作力。

　　切实转变工作作风。全面落实中央和省、市关于改进工作作风、密切联系群众的各项规定。多贴近基层、走进群众，多到矛盾和困难集中的地方去，多听群众中的真话实话，多为群众办实事好事。进一步开展市长市民面对面、恳谈日活动，尽心尽力为群众解决实际困难和问题。坚持厉行节约、勤俭办事，下基层轻车简从，简化接待，严禁铺张浪费。始终牢记“空谈误国、实干兴邦”的道理，改进会风文风，开短会、讲短话，去虚就实、去繁从简，集中精力干实事、破难题、促发展。

　　加强廉政建设。落实廉政建设责任制，认真贯彻执行廉洁从政各项规定，强化政府工作人员廉洁自律。完善责任审计制度，加强公共资源交易活动监管，继续强化项目投资评审，加大对重要领域、关键岗位的行政监察和审计监督力度，规范权力运行。加强对政府公职人员教育、管理和监督，努力打造一支为民务实清廉的公务员队伍。

　　各位代表！吉安正进入一个新的发展时期。美好的前景激励着我们，群众的期盼鞭策着我们。让我们紧密团结在以习近平同志为总书记的党中央周围，在省委、省政府和市委的正确领导下，深入贯彻落实党的十八大精神，进一步解放思想，团结拼搏，奋发进取，为建设开放繁荣秀美幸福新吉安，开启全面建成小康社会新征程而努力奋斗！
